# Supplementary material for: OpenMM 7: Rapid development of high performance algorithms for molecular dynamics
Source: PLoS Comput Biol. 2017 Jul 26;13(7):e1005659. doi: 10.1371/journal.pcbi.1005659 (PMC5549999; doi:10.1371/journal.pcbi.1005659)
Supplement: S1 Source Code — (ZIP) [file pcbi.1005659.s002.zip › OpenMM-7.0.1-Source/docs/api-c++/generated/OpenMM.Force.html]

Force — OpenMM @OPENMM\_MAJOR\_VERSION@.@OPENMM\_MINOR\_VERSION@ documentation


# Force¶

*class* `OpenMM::``Force`¶
:   `Force` objects apply forces to the particles in a `System`, or alter their behavior in other ways. This is an abstract class. Subclasses define particular forces.

    More specifically, a `Force` object can do any or all of the following:

    > - Add a contribution to the force on each particle
    > - Add a contribution to the potential energy of the `System`
    > - Modify the positions and velocities of particles at the start of each time step
    > - Define parameters which are stored in the `Context` and can be modified by the user
    > - Change the values of parameters defined by other `Force` objects at the start of each time step

    Forces may be organized into “force groups”. This is used for multiple time step integration, and allows subsets of the Forces in a `System` to be evaluated at different times. By default, all Forces are in group 0. Call `setForceGroup()` to change this. Some `Force` subclasses may provide additional methods to further split their computations into multiple groups. Be aware that particular Platforms may place restrictions on the use of force groups, such as requiring all nonbonded forces to be in the same group.

    Methods

    |  |  |
    | --- | --- |
    | `Force` |  |
    | `~Force` |  |
    | `getForceGroup` | Get the force group this `Force` belongs to. |
    | `setForceGroup` | Set the force group this `Force` belongs to. |
    | `usesPeriodicBoundaryConditions` | Returns whether or not this force makes use of periodic boundary conditions. |

    `Force`()¶

    `~Force`()¶

    int `getForceGroup`() *const*¶
    :   Get the force group this `Force` belongs to.

    void `setForceGroup`(int *group*)¶
    :   Set the force group this `Force` belongs to.

        |  |  |
        | --- | --- |
        | Parameters: |  |

        - **group** – the group index. Legal values are between 0 and 31 (inclusive).

    bool `usesPeriodicBoundaryConditions`() *const*¶
    :   Returns whether or not this force makes use of periodic boundary conditions. This method should be overridden for all `Force` subclasses, or a `OpenMM::OpenMMException` will be thrown

        |  |  |
        | --- | --- |
        | Returns: | true if `Force` uses periodic boundaries or false if it does not |

High performance molecular simulation on GPUs


### Navigation

- Core Objects
- Forces
- Integrators
- Extras

©2015, Stanford University and the Authors.
|
Powered by Sphinx 1.3.1
& Alabaster 0.7.7
|
Page source
